# Synchronization and phase between model cortical areas determine information transfer

**DOI:** 10.1186/1471-2202-14-S1-P387

**Published:** 2013-07-08

**Authors:** Marije ter Wal, Paul Tiesinga

**Affiliations:** 1 Department of Neuroinformatics, Donders Centre for Neuroscience, Radboud University Nijmegen, 6525 AJ Nijmegen, The Netherlands

## 

Oscillatory activity in the brain is often hypothesized to be a mechanism to flexibly establish a particular functional connectivity between brain areas. Transfer of information is thought to be related to high levels of synchronization and particular phase relations between areas in the brain (Communication Through Coherence, CTC). It is unclear under what conditions optimal synchronization and phase relations between brain areas are achieved and what constraints a coherence-based communication structure places on the electrophysiological properties and connectivity structure of the underlying local circuit. Here, we study a model network comprised of multiple local circuits, each representing a different cortical area, connected according to several different network topologies. We quantify the synchrony within and between the circuits and analyse the phase relations between the circuits. We study the effect of network coherence on the amount of information that can be transferred between areas and how this information is encoded.

We developed a model network of two interconnected local circuits. The circuits consisted of 100 fast-spiking interneurons and 400 pyramidal cells, connected by AMPA- and GABA-type synapses, with short axonal delays. The neurons were modelled as conductance-based point neurons. The connections between the neurons were made randomly, with connection probabilities that reflected anatomical data. The neurons received depolarizing input and an information-rich signal, consisting of correlated noise. Connections between circuits were purely excitatory. When the neurons are depolarized, the circuit shows oscillatory activity in the gamma frequency range (30-80 Hz). Frequency and synchrony of the circuit's oscillatory activity are determined by the level of depolarization.

In networks with unidirectional projections, we find that synchronization between areas is achieved when the receiving area oscillates at a lower frequency than the sending area and the projections are of sufficient strength to drive the receiver to increase its frequency. The highest level of synchrony is found when the receiving area is in a state of resonance. However, we find that high synchronization does not guaranty high information transfer, as quantified by the correlation between frequency modulations in the circuits across time. We show that information transfer is dependent on both high synchronization and a particular 'good' phase relation. The phase relation between areas can be altered by changing the depolarization of the receiver. In a bidirectional network we can distinguish between two conditions: 1) one circuit has a distinctly higher intrinsic frequency; 2) both circuits have similar intrinsic frequencies. Synchronization can occur in both conditions, though different constraints apply. If the two circuits have different intrinsic frequencies, the area with the highest frequency 'wins' and is the main sender of information in the network. Across conditions, information transfer is found to occur at high levels of synchronization, but only at particular phase relations between the areas. The optimal phase relation depends on the network state. These results are in line with recent experimental findings and indicate that a coherence-based network structure can underlie effective communication.

